# Dual Effects of Thermal Annealing on Rotomolded PLA Biocomposites: A Fiber-Content Study

**DOI:** 10.3390/polym18151802

**Published:** 2026-07-23

**Authors:** Erick Omar Cisneros-López, Josué Rivera-Aguilera, Rosa Gabriela López-Gonzaleznúñez, Pedro Ortega-Gudiño, Rubén González-Núñez, Esperanza González-Quezada, Roberto Carlos Vázquez-Fletes

**Affiliations:** 1 Centro Universitario de Ciencias Exactas e Ingenierías (CUCEI), Universidad de Guadalajara, Blvd. Gral. Marcelino García Barragán #1421, Guadalajara C.P. 44430, Mexico; rosa.lopez@academicos.udg.mx (R.G.L.-G.); pedro.ogudino@academicos.udg.mx (P.O.-G.); ruben.gnunez@academicos.udg.mx (R.G.-N.); esperanza.gonzalez@academicos.udg.mx (E.G.-Q.); 2 Departamento de Estado Sólido, Instituto de Física, Universidad Nacional Autónoma de México (UNAM), Ciudad de México C.P. 01000, Mexico; josue.rivera@unam.edu

**Keywords:** poly(lactic acid), rotational molding, thermal annealing, natural-fiber biocomposites, crystallinity, porosity, mechanical properties, water absorption, compost disintegration

## Abstract

Rotational molding is a shear-free technology to produce hollow plastic parts. Rotomolded poly(lactic acid) (PLA) and its biocomposites remain in a largely amorphous state, which limits their stiffness, toughness, and thermal resistance. Post-processing thermal annealing develops crystallinity without additives, but its combined effect with natural fibers has rarely been quantified. This work evaluates annealing at 100 °C for 1 h on rotomolded PLA biocomposites reinforced with 10, 20, and 30 wt.% of agave, coir, or pine fibers. Crystallinity (DSC, XRD), density and porosity, morphology (SEM), water absorption, mechanical properties (tensile, flexural, Charpy impact, Shore D hardness), and 28-day disintegration under lab-scale composting conditions were measured for treated and untreated samples. Annealing raised the matrix crystallinity from below 21% to 41–56% and produced predominantly α crystals with a nearly constant average size of about 20 nm. The treatment improved the matrix-dominated properties for every formulation: for neat PLA, Charpy impact strength increased by 120% (28.1 to 61.7 J/m), flexural strength by 53% (61.1 to 93.4 MPa), and flexural modulus from 3264 to 4511 MPa. Tensile strength and modulus, in contrast, remained unchanged or decreased. Porosity, set by fiber content, was unaffected by annealing; at 30 wt.%, the matrix barrier nonetheless reversed, and annealed samples absorbed more water and disintegrated faster than untreated ones. Fiber content sets the balance between matrix crystallinity and the interfacial damage caused by crystallization-induced contraction.

## 1. Introduction

Most of the global production of plastic materials ends its lifecycle in landfills, incineration sites, or natural environments [1,2], and annual production has continued to grow, while less than 10% of post-consumer plastic is recycled globally [2,3]. This low recycling rate has driven research on bio-based and biodegradable polymers within a circular economy of plastics [3,4]. Among these, poly(lactic acid) (PLA) is one of the most studied biopolymers. It comes from the fermentation of corn or sugarcane and is considered biodegradable under industrial composting conditions, and its degradation products show low toxicity [5,6]. The global PLA market has been growing at double-digit annual rates, with single-use packaging, agriculture, and biomedical devices as the main application drivers [7].

Despite these advantages, neat PLA presents two well-documented limitations that restrict its use in a wide range of structural and thermally demanding applications. The first is its inherent brittleness at room temperature, with elongations at break typically below 5% and low fracture toughness in comparison with conventional polyolefins [8]. The second is the slow natural crystallization rate of PLA during conventional cooling, which leaves most as-processed parts in a largely amorphous state and restricts their heat deflection temperature to values close to the glass transition near 60 °C [9,10]. These limitations are particularly relevant for processes that involve slow cooling stages and neither consolidation pressure nor shear stress, such as rotational molding [11,12].

Rotational molding is a versatile and cost-effective technology for the manufacture of seamless hollow parts with typical applications in water and chemical storage tanks, automotive components, sports equipment, and toys [12]. The low-shear and pressure-free nature of this process minimizes mechanical and thermal degradation of fibers and matrix, but it also restricts the development of crystallinity during the slow cooling stage and produces parts with higher porosity than compression or injection molding [13,14]. Greco and Maffezzoli [15] first reported the processing window of PLA by rotational molding, defining the conditions for proper sintering while avoiding thermal degradation. Cisneros-López et al. [16] later showed that the crystallinity of rotomolded PLA and PLA–agave fiber biocomposites reaches only 18%, less than half of the values achieved by compression molding under similar thermal cycles, confirming that the resulting parts exhibit reduced mechanical performance with increasing fiber content.

Natural fibers are a common reinforcement for PLA. They lower material cost, adjust stiffness, and keep the composite fully bio-based [17,18]. Agave, coir, and pine are particularly attractive as reinforcement in regional contexts due to their availability as agro-industrial residues, their low cost, and previous reports of their use in different processing routes [19,20]. The chemical composition of these three fibers differs substantially: agave and coir contain higher cellulose and lignin fractions, respectively, while pine is characterized by a high extractive content that has been shown to compromise the fiber–matrix interface in rotomolded systems [19]. To our knowledge, the combined effect of annealing and these three fibers in rotomolded PLA has not been previously reported.

Beyond composition, the inherent incompatibility between lignocellulosic fibers and the PLA matrix produces a weak fiber–matrix interface that limits stress transfer in the resulting biocomposites. This mismatch arises from the hydrophilic surface of the fibers and the hydrophobic character of PLA, which result in poor wettability and a low work of adhesion at the interface [19,21]. Moreover, depending on their surface chemistry, fillers may also influence the thermal degradation of the PLA matrix during processing, either accelerating or mitigating it [22]. All these considerations become particularly relevant in low-shear processes such as rotational molding, where the mechanical performance is consistently lower than that obtained by compression or injection molding [16]. To address this limitation, post-processing thermal annealing has been proposed as a simple and scalable route to develop crystallinity in PLA and biocomposite parts without the addition of nucleating agents or compatibilizers [23,24].

Several studies have quantified this effect in different processing routes. Yaisun and Trongsatitkul [23] reported that annealing at 120 °C for 30 min increased the crystallinity of compression-molded PLA hybrid composites of coir and bamboo leaf fibers, and raised the heat deflection temperature by 14%. Pérez-Fonseca et al. [24] applied thermal annealing at 100 °C for 40 min to rotomolded PLA–wood biocomposites and achieved crystallinity values up to 54%, with substantial improvements in impact strength and dimensional stability under accelerated weathering. Recent works have further confirmed that annealing close to the cold-crystallization temperature of PLA favors its thermodynamically stable α form over the disordered α′ modification, with a measurable impact on stiffness, heat resistance, barrier behavior, and resistance to hygrothermal aging [24,25,26,27,28,29]. The development of a denser crystalline phase, however, has also been associated with reduced ductility and lower tensile elongation in neat PLA and PLA blends [30,31], introducing a trade-off that has not been systematically explored in rotomolded biocomposites reinforced with natural fibers. The combined effect of natural fibers and annealing in low-shear processes has been rarely quantified.

Annealing-induced crystallinity also influences the end-of-life behavior of PLA biocomposites, an aspect that has received less attention than the mechanical and thermal effects discussed above. The dependence of PLA hydrolysis on its crystalline fraction is well established at the molecular scale [32], but its translation into the disintegration kinetics of rotomolded biocomposites under standardized composting conditions has been rarely addressed in a systematic manner. Recent studies have applied the ISO 20200:2023 [33] protocol to compostable PLA-based products and confirmed that crystallinity, fiber content, and interface quality govern the rate of mass loss [34].

Plant fibers accelerate the disintegration of PLA by promoting matrix swelling and creating preferential paths for microbial attack [35], while crystallinity slows down the hydrolysis of the amorphous fraction, which is the initial step of the disintegration process [35,36]. The balance between these two competing effects in rotomolded PLA biocomposites subjected to thermal annealing has, to our knowledge, not been previously reported. Understanding this balance is necessary to anticipate the end-of-life behavior of these materials.

This work evaluates the effect of post-processing thermal annealing at 100 °C for 1 h on PLA biocomposites reinforced with 10, 20, and 30 wt.% of agave, coir, or pine fibers, prepared by dry blending and rotational molding. The crystallinity (DSC, XRD), water absorption, mechanical performance (tensile, flexural, Charpy impact, Shore D hardness), and 28-day disintegration under lab-scale industrial composting conditions are systematically reported for treated and untreated samples as a function of fiber type and content, in order to quantify how fiber selection and fiber content modulate the effect of post-processing annealing on the resulting properties.

## 2. Materials and Methods

### 2.1. Materials

**Figure 1:**
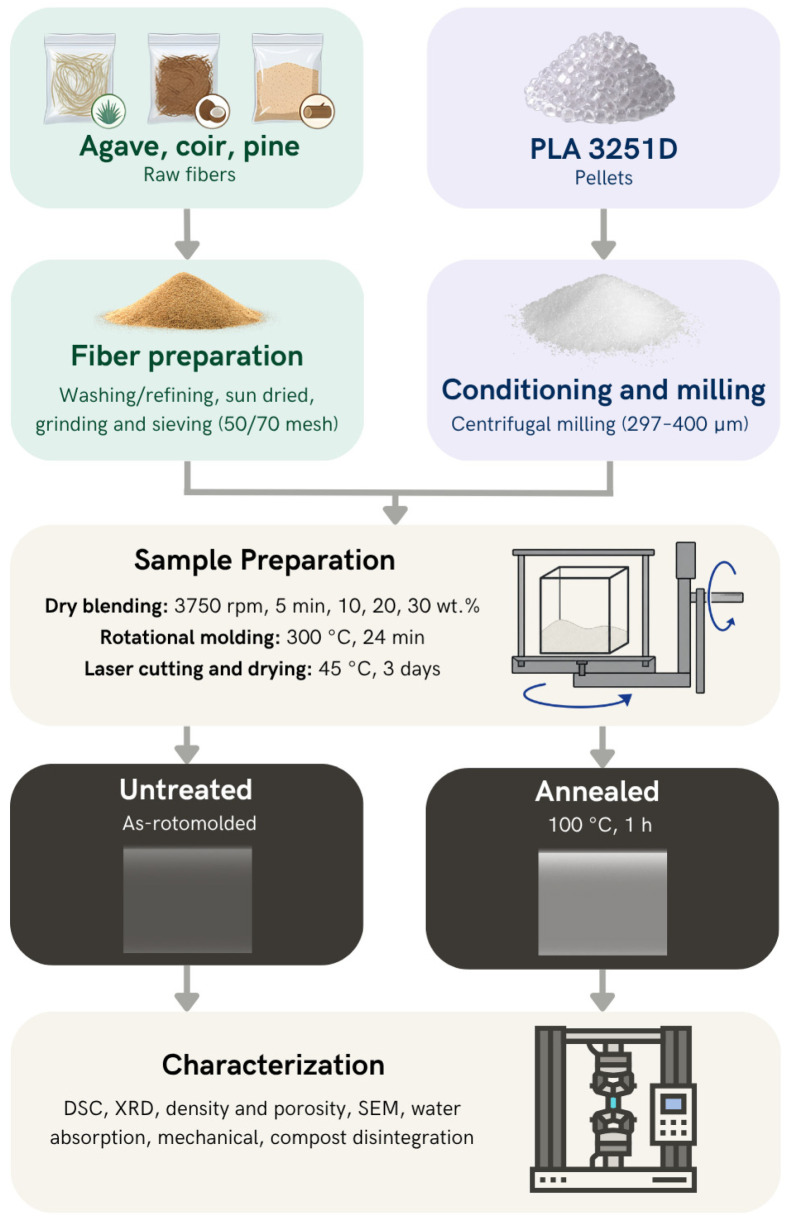
Processing route of the rotomolded PLA biocomposites, from fiber and matrix preparation to dry blending, rotational molding, and thermal annealing.

Poly(lactic acid) (PLA 3251D, NatureWorks LLC, Plymouth, MN, USA) was supplied by PromaPlast S.A. de C.V. (Guadalajara, Mexico) as injection-molding-grade pellets, with a melt flow index of 35 g/10 min (190 °C, 2.16 kg) and a density of 1.24 g/cm^3^. Three lignocellulosic fibers were used as reinforcement: agave fibers (*Agave tequilana* Weber var. Azul) from a local tequila distillery (Tequila, Mexico); coir fibers (*Cocos nucifera*) supplied by Agrocoir S.A. de C.V. (Colima, Mexico); and pine sawdust (*Pinus* spp.) supplied by Aserradero El Sur de Jalisco (Gómez Farías, Mexico). Figure 1 summarizes the processing route followed to produce the rotomolded biocomposites.

### 2.2. Fiber and Matrix Preparation

To remove dust, impurities, and soluble extractives, agave and coir fibers were immersed in water for 24 h at room temperature. Agave fibers were then passed through a Sprout-Waldron disk refiner (model D2A509NH, Sprout-Waldron, Muncy, PA, USA) to remove the residual pith and separate the individual fibers, using two 30 cm diameter disks, one fixed and the other rotating at 1770 rpm. Coir fibers were washed manually in a stainless-steel tank. Finally, these two materials were centrifuged and then sun-dried for 48 h. Pine sawdust was used as received. The three fibers were then ground and sieved in a Ro-Tap RX-29 shaker (W.S. Tyler, Mentor, OH, USA), retaining the fraction that passed the 50-mesh screen and was collected on the 70-mesh screen (particles between 210 and 297 μm).

The PLA was pulverized to enable dry blending with the fibers: the pellets were first conditioned in an Arctiko ULTF 80 ultra-freezer (Arctiko A/S, Esbjerg, Denmark) at −80 °C for 48 h to facilitate brittle fracture, and then ground in a Retsch ZM 200 centrifugal mill (Retsch GmbH, Haan, Germany) at 18,000 rpm to a particle size distribution of 297–400 μm, following the procedure previously validated by Cisneros-López et al. [16].

### 2.3. Physicochemical Characterization of the Fibers

The chemical composition of the three fibers was determined according to TAPPI standards T 204 cm-97 for extractives [37] and T 222 om-98 for lignin [38], and the Jayme–Wise method for holocellulose [39]. The results correspond to our previous characterization of the same fibers [19]. The fibers were also analyzed by X-ray diffraction (XRD) on a PANalytical Empyrean diffractometer (Malvern PANalytical, Almelo, The Netherlands), using Cu-Kα radiation (λ = 1.5406 Å) at 40 kV and 30 mA. Patterns were recorded over a 2*θ* range of 5–50° with a step size of 0.026° and a counting time of 27.5 s per step. The crystallinity index (CrI) of the cellulose was estimated by the Segal method [40], as commonly applied to lignocellulosic fibers [41], according to Equation (1):

(1) $$CrI\;\left(\%\right)=\frac{I_{200}-I_{am}}{I_{200}}\;\times 100$$

where $I_{200}$ is the maximum intensity of the cellulose I (200) reflection near 22.5° (2*θ*) and $I_{am}$ is the minimum intensity of the amorphous scattering near 18° (2*θ*) [40,41]. This index is a semi-quantitative comparative parameter and was used only to compare the three fibers with each other.

The skeletal density of the fibers ($\rho_{f}$), taken as an approximation to their cell-wall density [42], was measured by gas pycnometry on a Quantachrome Ultrapyc 1200e (Anton Paar, Graz, Austria) using nitrogen as the displacement gas. Volumetric measurements were performed using approximately 2.5 g of each dried fiber, with six replicates per fiber.

### 2.4. Dry Blending

Dry blends of PLA and each fiber were prepared at three fiber concentrations (10, 20, and 30 wt.%) using a high-speed industrial blender (JR Torrey LP-12, Monterrey, Mexico) at 3750 rpm for 5 min, following a procedure previously validated for PLA–natural-fiber systems [16,19]. Both PLA powder and fibers were oven-dried at 60 °C for 48 h prior to mixing, and the resulting blends were oven-dried again at 60 °C for 48 h before molding.

### 2.5. Rotational Molding of Biocomposites

Rotomolding was carried out in a custom-built laboratory-scale biaxial machine (CUCEI, Universidad de Guadalajara, Guadalajara, Mexico) equipped with a cube-shaped stainless-steel mold (15 cm × 15 cm × 16 cm, 2 mm wall thickness). The internal mold surface was covered with a thin layer of CP-500 release agent (Poliformas Plásticas, Guadalajara, Mexico). A total mass of 350 g of the corresponding dry blend was loaded into the mold, yielding parts with an average wall thickness between 2 and 5 mm depending on fiber content. The mold was sealed and introduced into the oven at 300 °C for 24 min (heating cycle), reaching a peak internal air temperature of 210 °C, and then cooled by forced-air convection for 24 min (cooling cycle) until the internal air temperature dropped to approximately 40 °C. A rotational speed ratio [13] of 4:1 was used during the entire process, with the main axis rotating at 3.6 rpm and the secondary axis at 2.7 rpm [16,43]. After demolding, the parts were cut into specimens of the appropriate geometry using a laser cutting machine and dried in an oven at 45 °C for 3 days, below the glass transition temperature of PLA, to remove moisture without inducing crystallization.

### 2.6. Thermal Annealing

Thermal annealing was performed in a digital convection oven (Pro-Lab, Guadalajara, Mexico, model PRO1002499, 30 L capacity, ±0.5 °C fluctuation, 250 °C maximum). The cut specimens were placed on a preheated ceramic flat plate and exposed to 100 °C for 1 h. This temperature lies above the glass transition of PLA but below its melting temperature, allowing cold crystallization without thermal degradation [23,24]. The samples were then cooled slowly inside the oven to room temperature. For each formulation, the untreated and annealed specimens were cut from the same rotomolded part. Samples are labeled as PLA, A, C, or P for neat PLA, agave-, coir-, and pine-reinforced systems, respectively, followed by the fiber content in wt.% and a final suffix “-U” for as-rotomolded (untreated) or “-T” for thermally annealed samples. For example, A10-T denotes the agave biocomposite at 10 wt.% subjected to thermal annealing.

### 2.7. Thermal and Structural Characterization

The thermal properties of the rotomolded biocomposites were analyzed using differential scanning calorimetry (DSC) on a TA Instruments Q100 calorimeter (TA Instruments, New Castle, DE, USA) under nitrogen atmosphere (50 mL/min). Samples of 4–6 mg were heated from 30 to 180 °C at 10 °C/min. A single heating scan was used in order to capture the effect of thermal annealing on the as-processed crystallinity of the samples. The glass transition temperature (*T_g_*), cold-crystallization temperature (*T_cc_*), and melting temperature (*T_m_*) were recorded. The degree of crystallinity (*X_c_*) was calculated using Equation (2) [16]:

(2) $$X_{c}\left(\%\right)=\frac{\Delta H_{m}-\Delta H_{cc}}{\Delta H_{ref}}\;\times \frac{100}{w}$$

where Δ*H_m_* and Δ*H_cc_* are the enthalpies of fusion and cold crystallization, Δ*H_ref_* is the theoretical enthalpy of fusion of fully crystalline PLA (93 J/g) [16], and w is the weight fraction of PLA in the biocomposite.

XRD patterns of the rotomolded samples were collected on the same diffractometer and under the conditions described in Section 2.3. The percent crystallinity was estimated according to Doumeng et al. [44] using Equation (3):

(3) $$X_{c}\left(\%\right)=\frac{A_{c}}{A_{c}+A_{a}}\;\times 100$$

where *A_c_* is the crystalline area and *A*_a_ is the amorphous halo area. The average crystal size *L* was estimated from the Scherrer equation [45,46] using Equation (4):

(4) $$L=\frac{K\lambda }{\beta cos(\theta )}$$

where *K* is the shape factor, *λ* is the wavelength of the incident radiation (1.5406 Å), *β* is the full width at half maximum (FWHM) in radians, and *θ* is the Bragg angle. Values of *K* of 0.94 and 0.90 were used for PLA and cellulose, respectively. For the biocomposites, an effective *K* was estimated as a weight-fraction-weighted average (Table 1), assuming that the fibers contribute as pure cellulose; this approximation is justified by the predominantly amorphous nature of hemicellulose and lignin in the relevant 2*θ* range. All data processing was performed in Python 3.13.5 (Python Software Foundation, Beaverton, OR, USA) using the NumPy 2.1.3, SciPy 1.15.3, and Matplotlib 3.10.0 libraries within the Spyder 6.1.0 integrated development environment. Integration of the DSC thermograms, deconvolution of the XRD diffractograms, determination of the FWHM, non-linear fitting of the Carter–Kibler model (Section 2.10), and the Pearson correlation analysis between water absorption and compost disintegration were carried out with these tools.

### 2.8. Density and Porosity

The skeletal density of the PLA ($\rho_{PLA}$) was measured by gas pycnometry under the same conditions described in Section 2.3, in both the untreated (U) and annealed (T) conditions, with four replicates per condition. Measurements were performed only on the pure components, i.e., the three fibers and the PLA. The skeletal density of the biocomposites was not measured directly, because the displacement gas does not access closed pores and would therefore include them in the solid volume, leading to an underestimation of the porosity. For that reason, the theoretical density ($\rho_{th}$) of each biocomposite was calculated by the rule of mixtures [47,48], from Equation (5):

(5) $$\frac{1}{\rho_{th}}=\frac{w}{\rho_{PLA}}\;+\frac{1-w}{\rho_{f}}$$

where w is the weight fraction of PLA and $\rho_{PLA}$ is its corresponding skeletal density (untreated or annealed). As defined in Section 2.3, $\rho_{f}$ represents the skeletal density of the fibers. This approach yields the density of the pore-free solid and allows the total porosity to be quantified [47].

The bulk density ${(\rho }_{b})$ was determined geometrically from the mass and the linear dimensions (length, width, and thickness) of regular rectangular specimens cut from the rotomolded parts, using a digital micrometer (Mitutoyo, Kawasaki, Japan) and a precision balance (Mettler Toledo, Greifensee, Switzerland). Liquid displacement methods (ASTM D792-20 [49]) were not used, because the lignocellulosic fibers absorb water and the high open porosity of the specimens allows the liquid to penetrate them. Three specimens were measured per formulation. The porosity was then obtained from Equation (6) [42,48]:

(6) $$Porosity(\%)=\left(1-\frac{\rho_{b}}{\rho_{th}}\right)\times 100$$

where $\rho_{b}$ is the individual value for bulk density of each specimen, and $\rho_{th}$ is the theoretical density of each formulation calculated from Equation (5).

### 2.9. Morphology

The morphology of the biocomposites was examined by scanning electron microscopy (SEM) on cryogenically fractured surfaces. Specimens were immersed in liquid nitrogen for at least 5 min and then fractured. The fragments were mounted on aluminum stubs with carbon tape and coated with gold for 30 s under vacuum in an SPI Module sputter coater (SPI Supplies, West Chester, PA, USA). Micrographs were collected on a Hitachi TM-1000 tabletop microscope (Hitachi High-Technologies, Tokyo, Japan), operated at a fixed accelerating voltage of 15 kV with a backscattered electron detector. Untreated and annealed samples of the same formulation were coated and observed in the same session. Magnifications between 50× and 400× were used, and the scale bar is indicated in each micrograph.

The macroscopic appearance of the rotomolded specimens was recorded by digital photography. The inner surface of the annealed biocomposites at 10, 20, and 30 wt.% of each fiber, and of the neat PLA in both the untreated and annealed conditions, was photographed under diffuse illumination against a matte black background, with a metric rule as a scale reference.

### 2.10. Water Absorption

Water absorption was evaluated on all formulations according to ASTM D570-22 [50], following a protocol adapted from our previous work on rotomolded PLA biocomposites [20]. Specimens were dried to constant weight, immersed in distilled water at room temperature, and weighed at 2 h, 24 h, 7 d, 14 d, 21 d, and 28 d after gentle surface drying. Five replicates per formulation were used. The data of neat PLA were fitted to the Carter–Kibler diffusion model [51] using Equation (7):

(7) $$M_{t}=\;(1\;-\frac{\beta }{\left(\beta \;+\;\alpha \right)}e^{-\alpha t}\;-\;\frac{\alpha }{\left(\beta \;+\;\alpha \right)}\frac{8}{\pi^{2}}e^{\frac{-D\pi^{2}t}{l^{2}}})M_{\infty }$$

where *M_t_* is the mass uptake at time *t*, *M*_∞_ is the equilibrium mass uptake, *D* is the water diffusion coefficient, *l* is the sample thickness, and *α* and *β* are the probabilities of bound water molecules becoming free and of free molecules becoming bound, respectively.

### 2.11. Mechanical Properties

Three-point flexural tests were performed on an Instron 3345 universal testing machine (Instron, Norwood, MA, USA) equipped with a 1 kN load cell at a crosshead speed of 2 mm/min and a span-to-depth ratio of 16 (ASTM D790-17 [52]). Tensile tests were performed on the same machine at a crosshead speed of 1 mm/min for Type V specimens (ASTM D638-14 [53]). At least five specimens per formulation were tested. Charpy impact strength was determined on notched specimens (ASTM D6110-18 [54]) using an Instron CEAST 9050 impact tester (Pianezza, Italy); eight specimens per formulation were notched with an Instron CEAST 6897 manual notcher (Pianezza, Italy) at least 24 h prior to testing. Shore D hardness (ASTM D2240-15 [55]) was measured with a Titanium 0–90 HD durometer (MainCasa, Guadalajara, Mexico); the reported values are the average of at least eight measurements.

### 2.12. Disintegration Under Simulated Composting Conditions

Disintegration tests were carried out according to ISO 20200:2023 [33]. A synthetic solid matrix simulating the organic fraction of municipal waste was prepared by mixing sawdust, rabbit feed, mature compost, corn starch, sucrose, corn-seed oil, urea, and distilled water in the proportions specified in the standard. Specimens of known initial dry mass were buried in plastic chambers containing the simulated compost and incubated at 58 ± 2 °C with a moisture content of 50 ± 5 wt.%. The matrix was mixed periodically, and water was added to compensate for evaporation losses. Specimens were retrieved at 7, 14, 21, and 28 days, gently rinsed with distilled water, oven-dried at 50 °C to constant mass, and weighed. Four replicates per condition were used. The percentage mass loss was calculated using Equation (8):

(8) $$Mass\;loss\;\left(\%\right)=\frac{m_{0}-m_{t}}{m_{0}}\;\times 100$$

where *m*_0_ is the initial dry mass and *m_t_* is the dry mass after each retrieval.

## 3. Results and Discussion

### 3.1. Physicochemical Properties of Fiber

Table 2 summarizes the chemical composition, the crystallinity index (*CrI*), and the skeletal density of the three fibers. The fibers differ mainly in their lignin and extractive contents. Coir has the highest lignin content (37–41 wt.%) and agave the highest holocellulose content (68–75 wt.%). In contrast, pine contains 18–20 wt.% of extractives, around three times the content of agave and coir. This difference is relevant for rotational molding, because the process is shear-free and the melted matrix must wet the fiber surface without mechanical compounding. In our previous work with these same fibers, the high extractive content of pine hindered the deposition of a maleated polyethylene coating on the fiber surface, whereas agave and coir responded to the same treatment [19].

**Figure 2:**
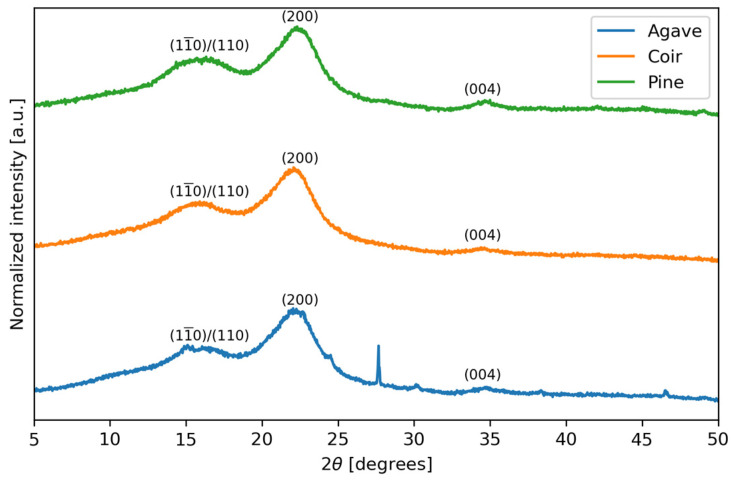
XRD diffractograms of the agave, coir, and pine fibers.

Figure 2 shows the diffractograms of the three fibers. All of them display the reflections of cellulose I: an unresolved signal near 16° (2*θ*) and the dominant (200) reflection near 22.5°. The crystallinity index calculated from Equation (1) is 42.3% for agave, 40.4% for coir, and 40.6% for pine. The three values fall within 2% of each other. The crystalline fraction of the cellulose is therefore comparable in the three fibers, and any difference in their interaction with the matrix would have to be associated with their chemical composition and/or morphology rather than with the crystallinity of their cellulose. The position of these reflections also matters for the analysis of the matrix: the (200) reflection of cellulose I near 22.5° coincides with the (210) reflection of α-PLA, and the signal near 16° overlaps with the dominant (110)/(200) reflection of PLA at 16.5° (Section 3.2).

The skeletal density measured by gas pycnometry is 1.420 ± 0.009 g/cm^3^ for agave, 1.425 ± 0.003 g/cm^3^ for coir, and 1.406 ± 0.003 g/cm^3^ for pine. The three values fall within 1.4% of each other, consistent with a cell wall dominated by the same lignocellulosic framework [42]. The value for agave matches the 1.426 g/cm^3^ measured for the same fiber in our previous work [56], and the value for pine agrees with the density reported for *Pinus sylvestris* particles determined by an independent method [47]. These densities are used in Equation (5) to calculate the theoretical density of each biocomposite.

### 3.2. Crystallinity

The combined effect of fiber type, fiber content, and post-processing thermal annealing on the crystallinity of the rotomolded biocomposites was assessed by DSC and XRD. DSC provides the enthalpic fraction of the matrix that crystallizes and melts during a single heating scan, while XRD captures the geometric fraction of long-range order present at room temperature [44]. The two techniques are not strictly equivalent; however, in semicrystalline PLA, they have repeatedly been shown to yield comparable results when the amorphous halo is consistently deconvoluted [23]. Therefore, both methods are reported, and their agreement was used as a cross-validation of the crystallinity values.

**Figure 3:**
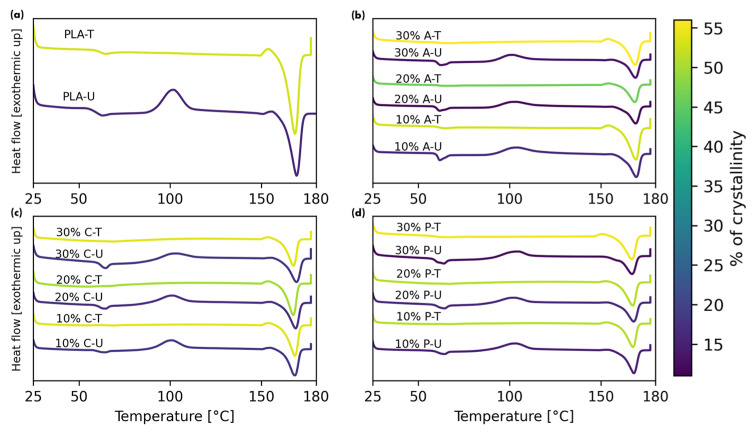
DSC thermograms of rotomolded PLA biocomposites, for untreated (U) and annealed (T) samples: (**a**) PLA, (**b**) agave, (**c**) coir, and (**d**) pine systems. Color scale encodes the DSC-derived crystallinity (*X_c_*).

Figure 3 presents the DSC thermograms grouped by fiber type. Curves are color-coded by the DSC-derived crystallinity of each sample. In all U curves, the exothermic cold-crystallization hump dominates the 90–110 °C interval and indicates that the matrix has insufficient time to crystallize during the cooling stage of the rotomolding cycle. In the T curves, this hump has been eliminated; only the glass transition step and the melting endotherm remain. The conversion of the cold-crystallization enthalpy into stable crystalline content is therefore visually unambiguous and consistent across the three fibers. A similar suppression of the cold-crystallization exotherm has been reported for compression-molded PLA–coir/bamboo composites annealed at 120 °C for 30 min [23], and for rotomolded PLA–wood biocomposites annealed at 100 °C for 40 min [24].

Table 3 shows the thermal parameters obtained from DSC. Untreated samples exhibit the three transitions typical of slow-cooled PLA: a glass transition near 60 °C, a cold-crystallization exotherm in the 101–104 °C range, and a melting endotherm at 167–169 °C. The presence of a well-defined cold-crystallization peak in every U sample (Δ*H_cc_* from 17.7 to 28.4 J/g) confirms that the rotational molding cycle leaves the matrix in a largely under-crystallized state, since the low-shear and pressure-free nature of the process prevents the development of crystallinity during cooling [15,16]. After annealing at 100 °C for 1 h, the cold-crystallization exotherm disappears in every formulation (Δ*H_cc_* = 0), which is direct evidence that the solid-state crystallization of the matrix was driven to completion during the thermal treatment. A slight increase in *T_g_* of up to 1.5 °C was observed in most annealed samples, which may be associated with the reduced mobility of the remaining amorphous phase due to the formation of crystalline regions [57].

The melting temperature is essentially conserved across all samples. *T_m_* stays within 167–169 °C, with differences below 2 °C between U and T counterparts. These values match those reported for α-PLA formed at moderate crystallization temperatures [57]. No bimodal melting endotherm and no low-temperature shoulder are observed, which indicates that the *α* form predominates in the annealed samples rather than the disordered α′ form typically obtained at lower annealing temperatures [28]. The crystallinity of the U samples ranges from 14.8% to 20.8%, in agreement with previous reports on rotomolded PLA biocomposites [16,24]. After annealing, *X_c_* rises into a narrow 41.4–55.9% range. The same crystallinity ceiling is observed for every formulation, independent of fiber type or content.

**Figure 4:**
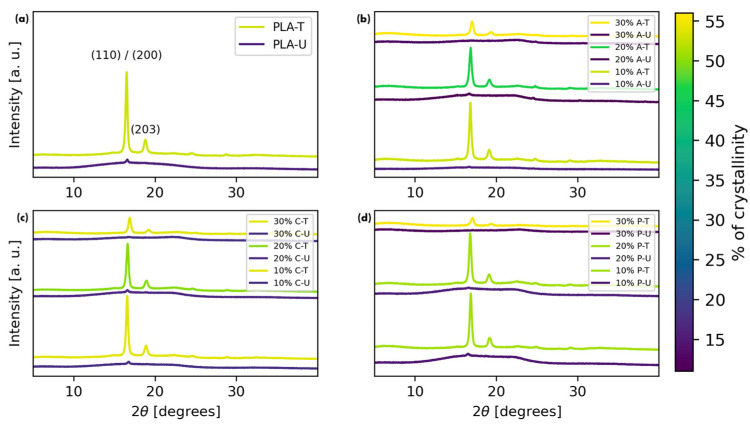
XRD diffractograms of rotomolded PLA biocomposites, for untreated (U) and annealed (T) samples: (**a**) PLA, (**b**) agave, (**c**) coir, and (**d**) pine systems. Color scale encodes the DSC-derived crystallinity *X_c_.*

Figure 4 reports the XRD diffractograms of U and T samples. The XRD patterns confirm the results from DSC. All annealed samples display the three reflections characteristic of α-PLA at 2*θ* around 16.5° (110/200), 18.9° (203), and 22.5° (210) [27], with intensities that increase abruptly compared with their U counterparts. Table 4 shows the XRD crystallinity results (*X_c_*) and the crystallite size (*L*) obtained from the Scherrer equation, Equation (4). For crystallinity, the results from XRD follow the same trend observed by DSC: the U samples fall in the 11.0–18.2% range, while the T samples are concentrated in the 46.3–56.2% range. The largest absolute deviation between DSC and XRD is observed for the agave-loaded systems (e.g., A20-U: 19.8 vs. 11.0%), which can be explained by the partial overlap of cellulose I reflections from the fiber with the dominant 16.5° peak of PLA [58], and by the lower signal-to-noise ratio of the diffractograms when most of the scattered intensity comes from the amorphous halo. For coir and pine biocomposites, the agreement between DSC and XRD is within ~3% for the annealed samples, supporting the robustness of the crystallinity measurements.

The crystallite size obtained from the Scherrer equation, Equation (4), provides a complementary view of the annealing process. In the U samples, the (110/200) reflection is weak and broad, which propagates as a large uncertainty in the FWHM fit. The *L* values for 30 wt.% U samples (4.0, 10.7 and 5.0 nm for agave, coir and pine, respectively) are particularly affected: the crystalline peak nearly vanishes, and the fit reflects the dominant amorphous halo rather than the small crystalline contribution. These values were reported only for completeness. After annealing, *L* falls into a much narrower 17.6–25.0 nm window across all formulations. Even though *X_c_* climbs by a factor of three to four during annealing, the average crystallite size remains essentially constant. *X_c_* and *L* are obtained independently: *X_c_* from the integrated crystalline area, Equation (3), and *L* from the peak breadth, Equation (4). The two quantities therefore separate the amount of crystalline phase from its characteristic size. A three- to four-fold rise in *X_c_* at constant *L* is consistent with an increase in the number of crystallites rather than in their size. This size is the coherent crystalline domain estimated from the Scherrer equation, not the spherulite dimension, so the data point to a higher crystallite number without resolving the spherulitic growth stage itself. Lignocellulosic surfaces have been reported to act as heterogeneous nucleation sites for PLA chains during annealing [23], consistent with the slightly smaller *L* observed for the 30 wt.% T samples (17.6–20.4 nm) compared with neat PLA-T (23.3 nm).

Taken together, DSC and XRD describe the same physical trend from two independent angles. The matrix leaves the rotomolding cycle with *X_c_* below 21% and a substantial cold-crystallization potential. Thermal annealing at 100 °C for 1 h produces predominantly *α* crystals and drives *X_c_* into the 41–56% range. The resulting population of crystallites has an essentially constant average size of ~20 nm regardless of fiber type or content. This crystalline state (particularly the *X_c_* values obtained from DSC) will be used as a starting point in the following subsections to interpret the water absorption, mechanical, and disintegration behavior of the biocomposites.

### 3.3. Density and Porosity of Biocomposites

**Figure 5:**
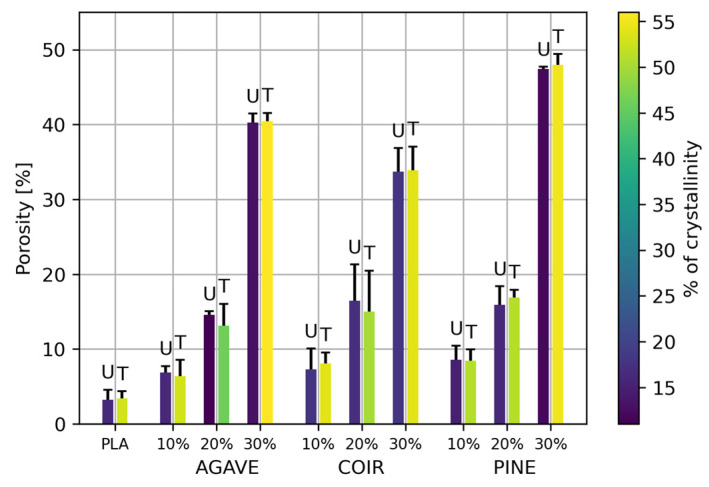
Porosity of the rotomolded biocomposites at 10, 20, and 30 wt.% of agave, coir, and pine, in the untreated (U) and annealed (T) conditions. Bars are color-coded by the DSC-derived crystallinity (*X_c_*) using the scale applied throughout the manuscript. Error bars represent the standard deviation (*n* = 3).

Table 5 presents the calculated theoretical density and results of bulk density, which led to estimation of the porosity of samples, presented in Figure 5. For neat PLA, the theoretical density reported in Table 5 is the skeletal density measured by gas pycnometry (Section 2.8), since Equation (5) reduces to the density of the matrix in the absence of fiber: 1.245 ± 0.004 g/cm^3^ for PLA-U and 1.252 ± 0.001 g/cm^3^ for PLA-T. The untreated value agrees with the 1.24 g/cm^3^ reported by the supplier for this grade (Section 2.1). Annealing raises the skeletal density by 0.6%, consistent with the crystallization of the amorphous phase measured by DSC and XRD. The skeletal density was therefore measured in both conditions rather than taken as a single value: using the untreated density for both, the densification of the matrix would be read as a closure of pores.

The bulk density shows an opposite pattern. It falls from 1.205 g/cm^3^ for neat PLA to 0.772, 0.858, and 0.678 g/cm^3^ at 30 wt.% of agave, coir, and pine, respectively. This occurs despite the three fibers being denser than the matrix (1.406–1.425 vs. 1.245 g/cm^3^, Table 2), so that the theoretical density rises over the same range while the measured one falls. The gap between the two is the porosity. This divergence originates in the process and not in the formulation: in our previous work with the same PLA–agave system, the skeletal density increased with fiber content regardless of the molding method, whereas the porosity of the rotomolded parts was consistently higher than that of the compression-molded ones, because no pressure acts on the melt and the two phases are never forced into contact [16]. The morphology of the resulting voids and of the fiber–matrix interface is examined in Section 3.4.

The porosity (presented in Figure 5) of neat PLA is 3.2%, and it increases with fiber content in the three systems: 6.4–8.6% at 10 wt.%, 13.1–16.9% at 20 wt.%, and 33.7–48.0% at 30 wt.%. The increase is not proportional to the fiber content. Between 20 and 30 wt.%, the porosity almost duplicates, and the bulk density falls below 0.9 g/cm^3^. As discussed earlier, in rotational molding, when fiber content rises, the melt must wet an increasing interfacial area through a denser network of particles, and the voids left between them are not closed [16].

As observed in Figure 5, annealing does not change the porosity. The ten formulations show the same value before and after the treatment, within the experimental error: 40.3 vs. 40.4% for A30, 33.7 vs. 33.9% for C30, and 47.4 vs. 48.0% for P30. This follows from the annealing conditions. At 100 °C, the material is far below the melting temperature of PLA (167–169 °C, Table 3), so it never flows, and the void structure formed during molding cannot be modified. As supported in Table 5, both the theoretical density and the bulk density of samples increase in the same proportion, and the calculated porosity is therefore unchanged.

The three fibers cannot be distinguished at 10 and 20 wt.%, where their porosities overlap within the standard deviation. They separate only at 30 wt.%: 47.4 ± 0.3% for pine, 40.3 ± 1.2% for agave, and 33.7 ± 3.2% for coir. This ranking follows the extractive content of the fibers (18–20, 6–7, and 4–6 wt.%, respectively; Table 2). Extractives are located at the fiber surface, and in our previous work with these same fibers, their high content in pine hindered the deposition of a coating on the fiber surface [56]. A surface that the melt does not wet leaves a void at the interface, and the effect becomes measurable at 30 wt.%, where the interfacial area is largest.

Porosity in these biocomposites is therefore governed by the fiber content and, at 30 wt.%, by the fiber type, but not by the thermal treatment. The property changes measured after annealing cannot be attributed to a change in the void content of the specimens.

### 3.4. Morphology of Biocomposites

**Figure 6:**
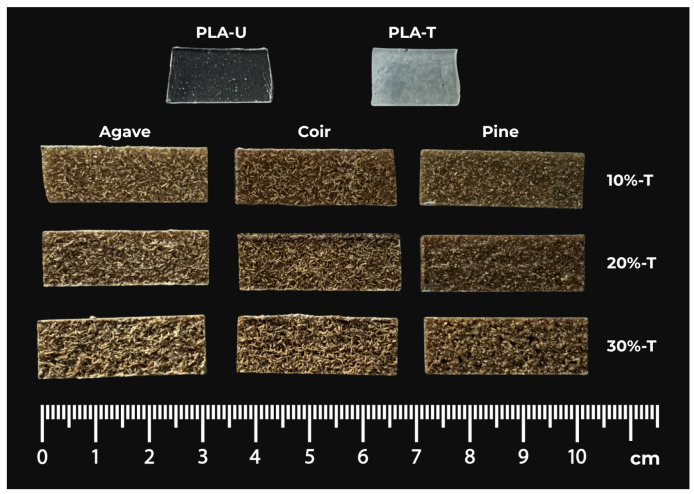
Macroscopic appearance of the inner surface of the rotomolded specimens. Columns correspond to agave, coir, and pine, and rows to 10, 20, and 30 wt.% fiber content. All biocomposites are shown in the annealed condition. Neat PLA is shown on the right in the untreated (U) and annealed (T) conditions. Neat PLA specimens are fragments of the rotomolded part.

Figure 6 shows the inner surface of the rotomolded specimens. Neat PLA-U is translucent and gray, while PLA-T is white and opaque. The whitening is the optical signature of crystallization and follows the crystallinity measured by DSC and XRD (Table 3, Section 3.2). The same change from a transparent to an opaque white specimen has been commonly reported for PLA annealed at 100 °C [59]. The biocomposites are opaque and brown at every fiber content, so the optical change may not be clearly discernible once fibers are present.

In rotational molding, the outer surface of the part replicates the mold wall and remains smooth, whereas the inner surface is not homogeneous and fibers are exposed on it [43]. Also, the absence of pressure on the melt limits compaction and contact between the phases and produces gaps at the surface [16]. The inner surface (reported in Figure 6) is then the surface on which defects are most visible. The proportion of the inner surface occupied by exposed fiber increases with fiber content. At 10 wt.%, the fibers appear as separated fragments in a continuous matrix. At 30 wt.%, the surface is fiber-dominated in the three systems.

**Figure 7:**
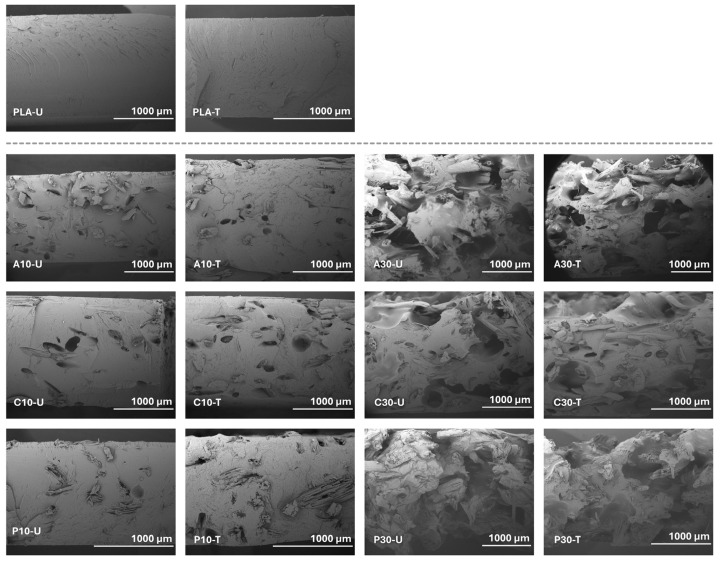
Cryo-fractured wall profiles of rotomolded neat PLA and PLA biocomposites, in the untreated (U) and annealed (T) conditions. Top row: neat PLA. Lower rows: agave, coir, and pine at 10 and 30 wt.% fiber loading (columns: 10-U, 10-T, 30-U, 30-T). Scale bars: 1000 µm.

Figure 7 shows cryo-fractured wall profiles of neat PLA and of the biocomposites at 10 and 30 wt.% fiber loading, in the untreated (U) and annealed (T) conditions. Neat PLA-U and PLA-T show a compact, uniform wall profile, with no difference resolved between the two conditions at this magnification. At 10 wt.%, the biocomposite profiles are compact, with dispersed fibers and few voids. At 30 wt.%, the profiles are open and irregular, with abundant fibers and large voids. The change from 10 to 30 wt.% is consistent across the three fibers. Pine at 30 wt.% shows the most disrupted profile, followed by agave, which agrees with the porosity ranking measured by gas pycnometry (pine 47.4%, agave 40.3%, coir 33.7%; Figure 5, Section 3.3). Agave and coir appear in cross-section as elongated fibers with a visible lumen, whereas pine appears as blocky, flattened particles. Fiber–matrix gaps that follow the fiber contour are present in both conditions and at both loadings.

**Figure 8:**
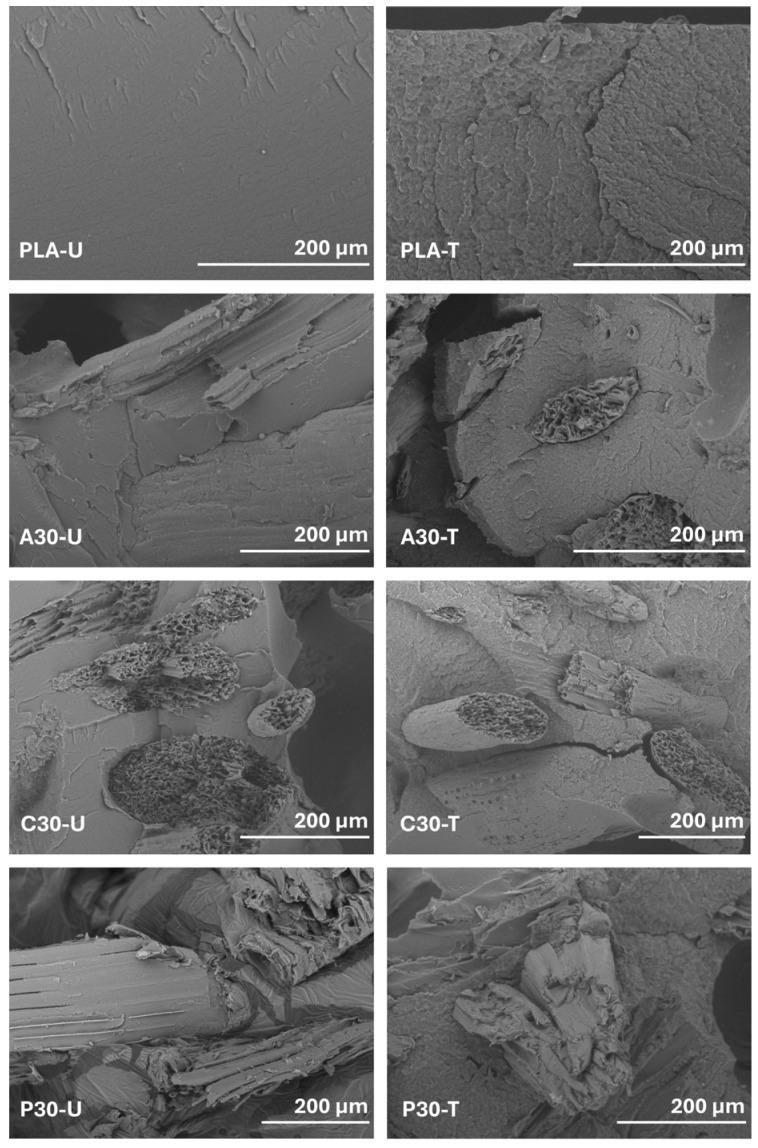
Cryo-fractured surfaces of rotomolded neat PLA and PLA biocomposites at 30 wt.% fiber loading, in the untreated (U) and annealed (T) conditions. Scale bars: 200 µm.

Figure 8 shows cryo-fractured surfaces of neat PLA and of the 30 wt.% biocomposites in the untreated (U) and annealed (T) conditions, at higher magnification. Neat PLA-U fractured with a smooth surface, with river patterns across the wall thickness, whereas PLA-T fractured with a granular surface. The granular features are 3–6 µm across and appear only in the annealed, crystalline specimens. The same change from a smooth to a rough cryo-fracture surface has been reported for PLA annealed at 100 °C over a comparable crystallinity range, and was attributed to the deformation of spherulites during fracture [59].

The biocomposites reproduce the matrix texture of the corresponding neat PLA condition: smooth in the untreated specimens and granular in the annealed ones. Fibers fractured transversely and left their lumens open; the coir bundles show the cellular structure of the fiber cross-section. Gaps between fiber and matrix are present in the three systems and in both conditions. These gaps are consistent with the poor wettability expected between the hydrophilic fibers and the hydrophobic matrix. This weak adhesion between untreated cellulosic fibers and PLA is documented for rotomolded parts [16,20]. Annealing does not create these gaps: they are already present in the untreated condition.

Matrix cracks are present in the annealed biocomposites and are absent in the untreated ones. The cracks appear in the crystallized matrix, where stresses at the fiber–matrix boundary from matrix shrinkage during crystallization have been reported to reduce fiber–matrix adhesion in PLA–natural-fiber composites [60]. Because a cryo-fracture surface cannot separate cracks formed during annealing from cracks formed during fracture, these cracks are read together with the mechanical, water uptake, and disintegration results and not on their own.

### 3.5. Water Absorption

Table 6 shows the water uptake of neat PLA and the biocomposites at all selected immersion times, for untreated (U) and annealed (T) samples. Three behaviors are identified from the data. Neat PLA absorbs less water after annealing. At 10 and 20 wt.% fiber loadings, treated and untreated samples reach similar overall uptake; at longer immersion times (above 14 d), the treated samples tend to show slightly higher values than the untreated ones, more clearly at 20 wt.%. At 30 wt.%, treated samples absorb substantially more water than their untreated counterparts. The three trends are observed for the three fiber types, which indicates that the underlying mechanism is governed by the matrix–fiber interaction rather than by differences in fiber chemistry.

**Figure 9:**
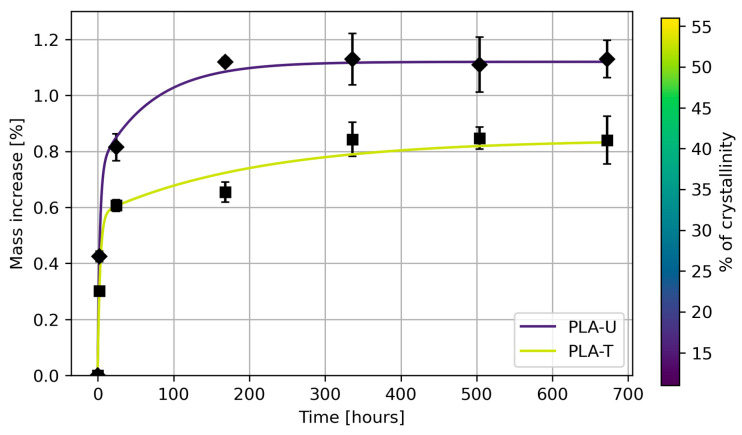
Water uptake of neat PLA as a function of immersion time. Untreated (PLA-U, ◆) and annealed (PLA-T, ■) samples. Symbols are experimental means (*n* = 5) with standard deviation bars; solid lines are the Carter–Kibler two-stage fit (Equation (7)) [51]. Colors follow the DSC-*X_c_* scale used throughout the manuscript.

The water uptake of neat PLA at 28 d decreases from 1.13% (PLA-U) to 0.84% (PLA-T). The Carter–Kibler [51] two-stage model (Equation (7)) was fitted to the PLA data; the resulting parameters are presented in Table 7, and the experimental points are shown together with the model curves in Figure 9. The fit presented values of *R*^2^ = 0.9997 and 0.9868 for PLA-U and PLA-T, respectively. The model was applied only to neat PLA because in the matrix alone, the water transport is governed solely by the crystalline state of the polymer, which is the variable modified by annealing. In the biocomposites, the absorption is dominated by the hydrophilic fiber phase and by the structural state of the fiber–matrix interface—two contributions whose individual effects cannot be uniquely extracted—yielding parameters that would be ambiguous and not directly comparable with the literature.

However, a closer look at the kinetics reveals subtle differences. At initial times (2–24 h), several treated samples take up less water than their untreated counterparts, which is consistent with the crystalline matrix barrier effect still operating at short times. At 20 wt.% and immersion times above 14 d, the trend reverses: T samples consistently show slightly higher water uptake than their U counterparts, suggesting that a second mechanism is already contributing at this fiber loading. As immersion proceeds, the hydrophilic nature of the lignocellulosic surfaces dominates the long-term equilibrium of the 10 wt.% systems [20,61], and the initial differences between treated and untreated samples are progressively erased. At 20 wt.%, the slight excess uptake of the T samples anticipates the larger effect observed at 30 wt.%, which is discussed below.

At 30 wt.%, the trend completely reverses. Annealed samples consistently absorb more water than their untreated counterparts at 28 d: 41.2 ± 0.8% (T) versus 25.8 ± 1.2% (U) for agave, 27.5 ± 1.6% (T) versus 20.1 ± 2.4% (U) for coir, and 37.8 ± 2.4% (T) versus 20.9 ± 1.2% (U) for pine. The effect is not subtle. The matrix barrier predicted from crystallinity is not only canceled but also reversed. The most plausible explanation is the volumetric contraction of the matrix during annealing-driven crystallization. As crystallinity develops during annealing, the PLA matrix densifies and its specific volume decreases [62]; this contraction takes place around fibers that do not deform. At 10 and 20 wt.%, the stress fields generated at each fiber–matrix interface are spatially isolated and can be accommodated elastically by the surrounding matrix. At 30 wt.%, the fibers are close enough for these fields to overlap, and the resulting interfacial stresses exceed the cohesive limit of the matrix. This interfacial damage, consistent with the cracks observed in the annealed composites (Section 3.4), opens additional capillary pathways for water ingress [62,63]. Once water reaches the now-exposed fibers, swelling further widens the gaps, and the process becomes self-reinforcing [64].

The water absorption results therefore reveal that annealing affects two distinct length scales in the biocomposites: the crystalline organization of the matrix and the structural state of the fiber–matrix interface. The two effects compete, and the balance between them depends primarily on fiber content. The mechanical response examined in Section 3.6 provides an independent probe of this competition, since the load transfer between matrix and fiber is itself controlled by the integrity of the interface.

### 3.6. Mechanical Properties

**Figure 10:**
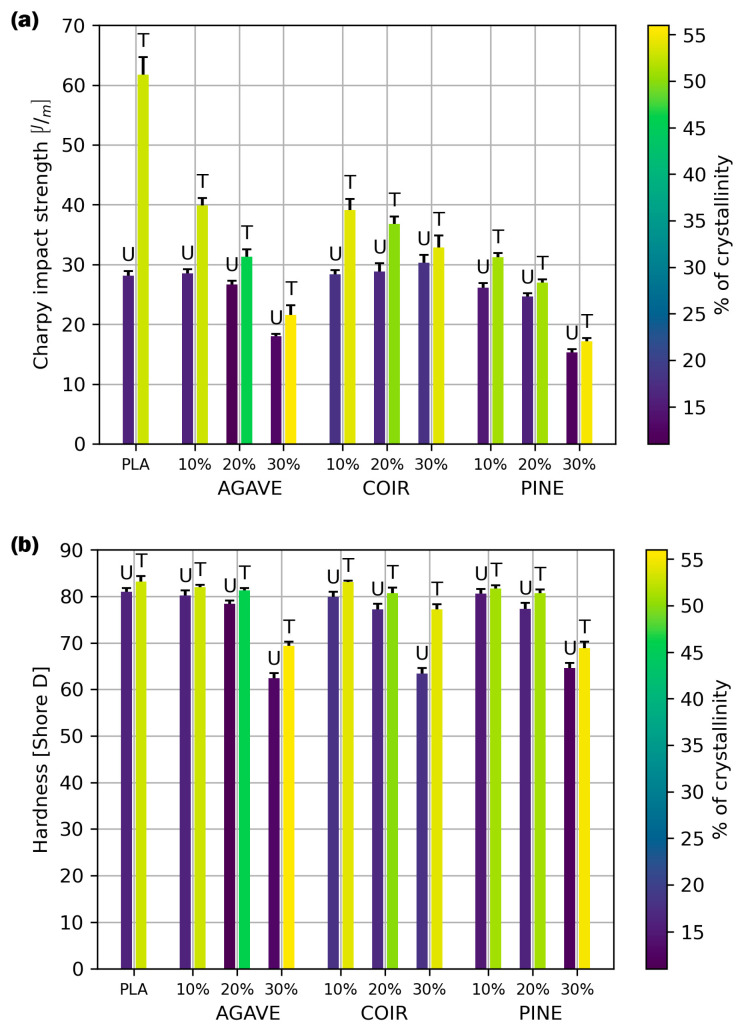
(**a**) Charpy impact strength and (**b**) Shore D hardness of neat PLA and the biocomposites, for untreated (U) and annealed (T) samples. Error bars represent the standard deviation. Bars are color-coded by the DSC-derived *X_c_* scale used throughout the manuscript.

**Figure 11:**
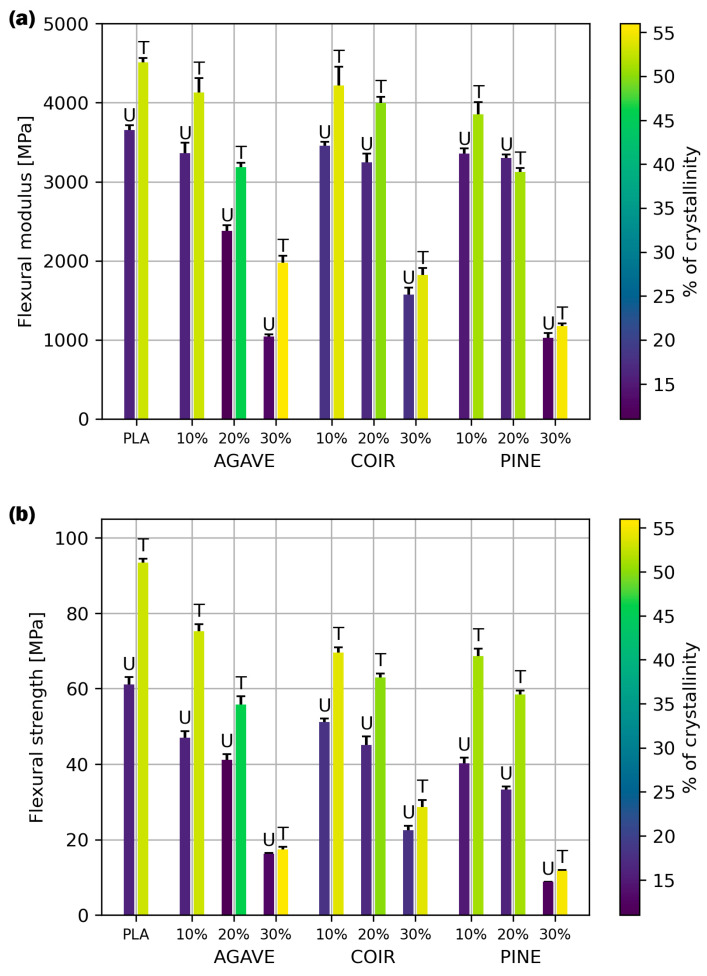
(**a**) Flexural modulus and (**b**) flexural strength of neat PLA and the biocomposites, for untreated (U) and annealed (T) samples. Error bars represent the standard deviation. Bars are color-coded by the DSC-derived *X_c_* scale used throughout the manuscript.

**Figure 12:**
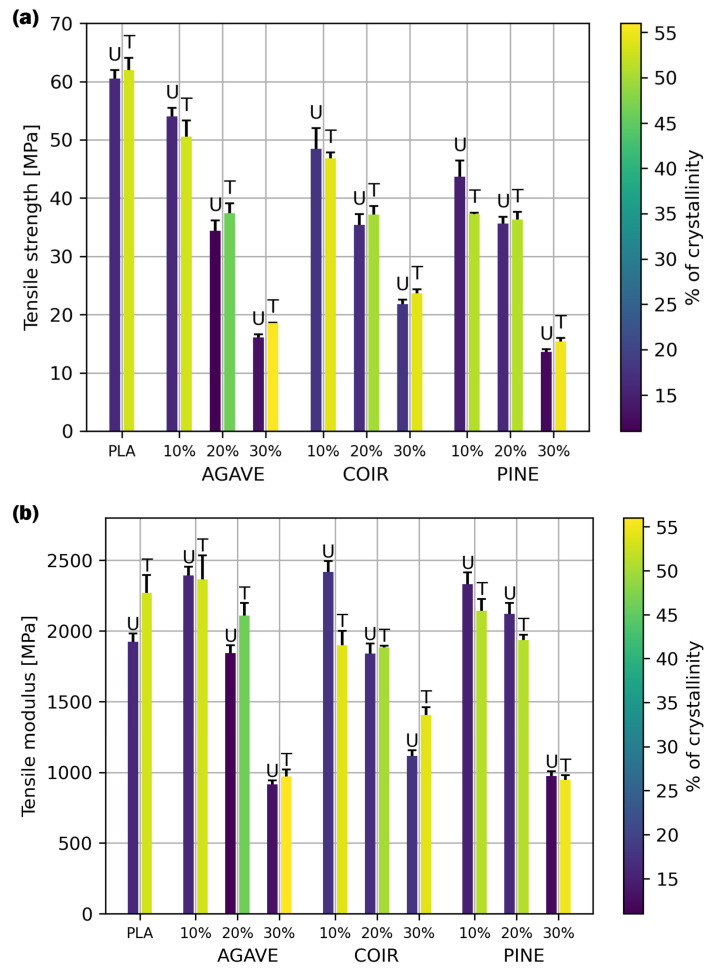
(**a**) Tensile strength and (**b**) tensile modulus of neat PLA and the biocomposites, for untreated (U) and annealed (T) samples. Error bars represent the standard deviation. Bars are color-coded by the DSC-derived *X_c_* scale used throughout the manuscript.

The mechanical response of the biocomposites is summarized in Figure 10, Figure 11 and Figure 12. Figure 10 displays the Charpy impact strength and Shore D hardness; Figure 11 presents the flexural modulus and flexural strength; and Figure 12 shows the tensile strength and tensile modulus. Values are presented as mean ± standard deviation and bars color-coded by the *X_c_* obtained from DSC (Section 3.2), so the visual scale is consistent across the manuscript. Two main behaviors are identified from mechanical characterization. The first three properties (Charpy impact, hardness, and flexural) improve systematically upon annealing for all fiber types and contents, in agreement with the increased crystallinity of the matrix. The tensile response, in contrast, follows a different trend: at the lowest fiber loadings, the annealed samples reach values comparable to or even lower than their untreated counterparts. This distinction between matrix-dominated and interface-sensitive properties is the central observation of this section.

Thermal annealing produced the largest mechanical change in the Charpy impact strength of neat PLA, which more than doubled (120%) from 28.1 ± 0.8 J/m in PLA-U to 61.7 ± 3.0 J/m in PLA-T (Figure 10a). The improvement persists in the biocomposites, although the magnitude decreases with fiber content: A10 increased from 28.6 to 39.9 J/m, C10 from 28.3 to 39.1 J/m, and P10 from 26.1 to 31.2 J/m. At 30 wt.%, the gain is smaller but still present in all fiber systems. The mechanism is consistent with PLA crystallites deflecting propagating cracks at spherulite boundaries, dissipating energy and delaying failure [65].

The Shore D hardness (Figure 10b) follows a parallel trend: T samples are systematically harder than U samples for all formulations, with differences of 1 to 7 points. The largest relative change occurs at 30 wt.%, where the annealed samples partially recover the hardness loss caused by the high fiber content (from 62.4 to 69.4 for A30, 63.4 to 77.2 for C30, and 64.6 to 68.9 for P30). Both properties confirm that the matrix becomes harder and tougher after annealing, consistent with the higher *X_c_* and with previous reports on annealed PLA [29] and rotomolded natural-fiber composites [19].

As observed in Figure 11, the flexural properties reinforce the same trend. The flexural modulus of neat PLA increased from 3264 to 4511 MPa after annealing (Figure 11a), and the gain extends to all biocomposites: A10 from 3361 to 4131 MPa, C10 from 3453 to 4217 MPa, and P10 from 3355 to 3851 MPa.

The flexural strength shows an even larger relative improvement (Figure 11b): PLA-T reaches 93.4 ± 1.1 MPa compared to 61.1 ± 1.9 MPa for PLA-U, a 53% increase. The biocomposites follow the same trend with consistent gains of 25 to 70% in the 10 and 20 wt.% systems. At 30 wt.% the absolute values decrease sharply for both U and T as a consequence of the high fiber content, but T samples remain above U samples in every case. Three-point flexure loads the specimen in a combination of compression on the loaded face and tension on the opposite face, with maximum stress at the outer surfaces. In composites with short, randomly oriented fibers, the matrix carries a large fraction of this load, so its integrity dominates the response, and the increased *X_c_* produced by annealing translates directly into higher stiffness and strength [66].

Tensile properties are presented in Figure 12. The tensile response departs from the previous pattern. In neat PLA, the tensile strength is essentially unchanged by annealing (60.5 ± 1.4 MPa for PLA-U and 61.9 ± 2.1 MPa for PLA-T), and the tensile modulus increases only modestly (1922 to 2268 MPa). More significantly, several biocomposites at low loadings show T values equal to or lower than U. Tensile strength decreases in A10 (54.0 ± 1.4 to 50.5 ± 2.8 MPa) and P10 (43.7 ± 2.8 to 37.3 ± 0.3 MPa). The same direction appears in tensile modulus for C10 (2418 to 1897 MPa), P10 (2330 to 2143 MPa), and P20 (2121 to 1934 MPa). The crystallinity-driven gains seen in Charpy and flexural performance do not translate into tensile gains at these loadings; in some cases, the opposite is observed. A similar trade-off between matrix- and interface-controlled properties has been reported in other PLA systems subjected to annealing: PLA blends in which crystallinity raises modulus and heat resistance while sharply reducing tensile ductility [30], and a PLA/magnesium composite in which annealing reduced its tensile strength [31].

Tensile testing loads the specimen in pure axial elongation, and the load transfer from matrix to fiber is primarily governed by the cohesion of the fiber–matrix interface. Any reduction in interfacial adhesion directly appears as a loss in tensile properties [61,63]. The volumetric contraction of the matrix during annealing-driven crystallization, already discussed in Section 3.5 to explain the excess water uptake at 30 wt.%, generates stress fields around the fibers that can damage the interface even at low fiber contents [63]. This interfacial damage is consistent with the cracks observed in the annealed composites (Section 3.4). Charpy, hardness, and flexural tests are less sensitive to this damage because they probe a stress state in which the matrix carries the dominant load.

The mechanical results define two categories of behavior. Charpy impact, Shore D hardness, and the two flexural properties are matrix-dominated: they improve with annealing because they reflect the bulk stiffness and toughness gained from the increased crystallinity. Tensile strength and tensile modulus are interface-sensitive: they depend on the cohesion of the fiber–matrix interface, a property that annealing simultaneously damages. As argued above, the volumetric contraction of the matrix during cold crystallization around non-deforming fibers explains the gains in matrix-dominated properties and the losses in interface-sensitive ones [62]. This trade-off is consistent with the water absorption results in Section 3.5 and predicts changes in the end-of-life behavior, which is examined next.

### 3.7. Disintegration

The disintegration of the biocomposites in laboratory-scale compost provides a third independent probe of the trade-off identified in Section 3.5 and Section 3.6. Table 8 lists the mass loss of all samples at 7, 14, 21 and 28 days. Three patterns are identified, governed by fiber content. Neat PLA disintegrates more slowly after annealing. At 28 days, PLA-T reached 35.7 ± 1.8% mass loss against 54.4 ± 2.9% for PLA-U, a 34% reduction. The result is consistent with the established role of α crystallinity as a barrier to hydrolytic and microbial attack on PLA [35,36]. The compact crystalline domains created during annealing reduce chain mobility and water diffusivity (as already quantified in Section 3.5) and slow the initial steps of hydrolysis.

At intermediate fiber loadings (10–20 wt.%), the response varies with fiber type. Coir composites still follow the matrix barrier pattern: C10-T (38.9 ± 2.3%) and C20-T (28.1 ± 2.8%) disintegrate slower than their untreated counterparts at 28 days (56.8 ± 5.9% for C10-U and 33.5 ± 1.3% for C20-U). Agave composites show comparable values at 10 wt.% but already exhibit acceleration at 20 wt.% (A20-T 69.6 ± 5.5% vs. A20-U 47.4 ± 1.4%). Pine composites accelerate at the lowest threshold: P10-T (54.2 ± 3.8%) exceeds P10-U (22.2 ± 0.5%) by a factor of 2.4. This trend is consistent with interfacial stress fields that build up with fiber content and breach the cohesive limit of the matrix only where they overlap.

As observed in water absorption and mechanical properties, at 30 wt.%, the trend reverses across all three fiber types. A30-T reaches 74.4 ± 5.7% at 28 days against 65.1 ± 4.6% for A30-U; C30-T reaches 55.6 ± 2.2% against 45.0 ± 3.1% for C30-U; and P30-T reaches 92.6 ± 2.9% at 14 days and 100% at 21 days, while P30-U only reaches 65.6 ± 0.1% at 28 days. The same mechanism invoked for the excess water uptake at 30 wt.% in Section 3.5 and for the tensile loss in Section 3.6 applies here: the volumetric contraction of the matrix during cold crystallization generates stress fields at the fiber–matrix interface that, at high fiber content, overlap and exceed the cohesive limit of the matrix [63]. The interfacial damage inferred at 30 wt.%, which already manifested as accelerated water uptake, provides preferential routes for moisture, oxygen and microorganisms in compost, and the disintegration kinetics jump accordingly [35].

**Figure 13:**
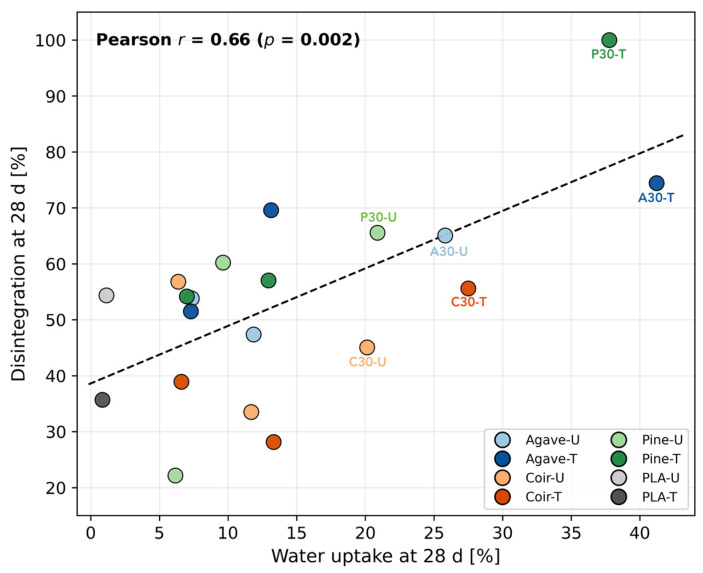
Correlation between 28-day water uptake and 28-day mass loss for all twenty formulations. Pearson *r* = 0.66 (*p* = 0.002). The dashed line is the least-squares linear fit and is shown only to indicate the trend.

The 28-day water uptake and the 28-day mass loss were correlated across all twenty formulations (Figure 13). The two variables are positively correlated (Pearson *r* = 0.66, *p* = 0.002): formulations that absorb more water also disintegrate faster. This trend is governed by the annealed composites, in which the same interfacial damage opens pathways for both water ingress and microbial attack. The clearest cases lie at 30 wt.%, where the annealed composites that absorb the most water (A30-T, P30-T, C30-T) are also among the fastest to disintegrate.

A third contribution cannot be ruled out. Beyond crystallinity and interfacial stress, the surface chemistry of a filler can influence the thermal degradation of the PLA matrix during processing, either accelerating or mitigating it depending on the filler–matrix interaction [22]. The lignocellulosic fibers used here were incorporated without surface treatment, so a filler-induced contribution to matrix degradation cannot be excluded and merits further study.

The disintegration results therefore reveal the third side of the same trade-off. Annealing protects the end-of-life behavior of neat PLA by slowing matrix hydrolysis but accelerates disintegration at high fiber contents through the same interfacial damage that drives the water absorption and tensile responses. The three independent characterizations (water uptake, mechanical response and compost disintegration) converge on the same observation: fiber content sets the balance between the protective effect of matrix crystallinity and the destabilizing effect of crystallization-induced interfacial stresses.

## 4. Conclusions

Post-processing thermal annealing at 100 °C for 1 h raised the crystallinity of rotomolded PLA biocomposites from below 21% to 41–56%, independently of fiber type and content. DSC and XRD both indicated that the treatment converts the cold-crystallization potential of the untreated as-rotomolded matrix into predominantly α crystals with an almost constant average size of about 20 nm. Because this size stays constant while crystallinity increases, the increase is consistent with a higher number of crystallites rather than larger ones.

The treatment had two opposite effects on the resulting properties. Matrix-dominated properties (Charpy impact, Shore D hardness, flexural strength and modulus) improved for every formulation, in agreement with the higher crystallinity. Interface-sensitive properties (tensile strength and modulus) remained unchanged or decreased, because the volumetric contraction of the matrix during crystallization damages the fiber–matrix interface. Gas pycnometry showed that the apparent porosity did not change with annealing, confirming that the treatment densifies the matrix rather than modifying the void structure formed during rotomolding. SEM of cryo-fractured surfaces was consistent with this interfacial damage, showing cracks in the annealed composites that were absent in their untreated counterparts.

Fiber content sets the balance between these two effects. At 10 and 20 wt.%, the matrix barrier dominated, and annealing reduced water absorption and slowed disintegration. At 30 wt.%, the trend reversed: annealed samples absorbed more water and disintegrated faster than their untreated counterparts. Water absorption, mechanical response, and compost disintegration converge on the same mechanism, in which crystallization-induced interfacial stress governs the behavior of the biocomposites at high fiber content. Thermal annealing is therefore a viable strategy to tailor the properties of rotomolded biocomposites. However, composition and treatment must be designed together in order to obtain materials suited to a specific application and a defined end-of-life scenario.

## Figures and Tables

**Table 1:** Effective Scherrer shape factor (*K*) used to estimate the average crystal size of the rotomolded biocomposites. The values were calculated as a weight-fraction-weighted average of *K* = 0.94 for PLA and *K* = 0.90 for cellulose.

Fiber Content (wt.%)	0	10	20	30
*K*	0.940	0.936	0.932	0.928

**Table 2:** Chemical composition, crystallinity index (*CrI*), and skeletal density (*ρ_s_*) of the agave, coir, and pine fibers. Composition values correspond to our previous characterization of the same fibers [19]. Skeletal density is expressed as mean ± standard deviation (*n* = 6).

Fiber	Extractives (wt.%)	Lignin (wt.%)	Holocellulose (wt.%)	*CrI* (%)	*ρ_s_* (g/cm^3^)
Agave	6–7	21–24	68–75	42.3	1.420 ± 0.009
Coir	4–6	37–41	58–61	40.4	1.425 ± 0.003
Pine	18–20	27–30	52–60	40.6	1.406 ± 0.003

**Table 3:** Thermal parameters and degree of crystallinity of rotomolded PLA biocomposites reinforced with 10, 20 or 30 wt.% of agave (A), coir (C) or pine (P) fibers, for untreated (U) and annealed (T) samples, obtained from a single DSC heating scan.

Sample	*T_g_* (°C)	*T_cc_* (°C)	*T_m_* (°C)	Δ*H_cc_* (J/g)	Δ*H_m_* (J/g)	*X_c_* (%)
PLA-U	60.4	101.6	169.3	28.4	42.2	14.8
PLA-T	61.9	—	168.4	—	43.4	46.7
A10-U	60.4	104.2	169.4	20.6	37.9	20.6
A10-T	61.4	—	169.1	—	38.8	46.3
A20-U	60.2	103.3	169.1	21.1	35.8	19.8
A20-T	61.2	—	168.6	—	30.8	41.4
A30-U	60.5	101.4	168.9	21.7	31.4	14.9
A30-T	61.1	—	168.8	—	30.7	47.1
C10-U	60.0	101.0	168.3	23.8	40.7	20.2
C10-T	60.1	—	168.4	—	46.0	55.0
C20-U	61.0	101.6	168.8	24.6	38.5	18.7
C20-T	60.9	—	167.5	—	39.3	52.8
C30-U	61.4	103.7	169.3	17.7	31.2	20.8
C30-T	61.4	—	167.6	—	36.4	55.9
P10-U	60.5	103.4	168.1	24.6	40.2	18.7
P10-T	59.9	—	167.6	—	42.7	51.0
P20-U	60.5	102.7	168.0	20.3	34.4	19.0
P20-T	60.7	—	167.5	—	37.9	50.9
P30-U	59.6	104.0	167.2	17.7	28.4	16.5
P30-T	59.9	—	167.0	—	36.2	55.6

**Table 4:** Average crystallite size (*L*), crystalline area (*A_c_*), amorphous halo area (*A_a_*), and degree of crystallinity (*X_c_*) of rotomolded PLA biocomposites, for untreated (U) and annealed (T) samples, obtained from XRD.

Sample	*L* (nm)	*A_c_* (Counts⋅°)	*A_a_* (Counts⋅°)	*X_c_* (%)
PLA-U	31.9	5460	27,869	16.4
PLA-T	23.3	25,095	22,369	52.9
A10-U	28.3	5544	30,078	15.6
A10-T	23.7	13,375	11,967	52.8
A20-U	23.0	3177	25,809	11.0
A20-T	20.9	8425	9775	46.3
A30-U	4.0 *	1297	8633	13.1
A30-T	19.9	4717	3669	56.2
C10-U	30.7	4186	18,868	18.2
C10-T	25.0	23,469	20,220	53.7
C20-U	27.3	5523	26,615	17.2
C20-T	22.9	18,957	18,894	50.1
C30-U	10.7 *	2819	13,204	17.6
C30-T	20.4	8026	6938	53.6
P10-U	28.8	5896	33,152	15.1
P10-T	23.5	14,379	13,711	51.2
P20-U	27.3	4537	24,604	15.6
P20-T	22.4	14,449	13,960	50.9
P30-U	5.0 *	757	5803	11.5
P30-T	17.6	3050	2509	54.9

* Values for which the (110/200) reflection nearly vanishes and the FWHM fit is not physically reliable; reported only for completeness.

**Table 5:** Theoretical density ($\rho_{th}$) and bulk density ($\rho_{b}$) of the untreated (U) and annealed (T) rotomolded specimens. Values are expressed as mean ± standard deviation (*n* = 3).

Sample	$\rho_{th}$ (g/cm^3^)	$\rho_{b}$ (g/cm^3^)
PLA-U	1.245	1.205 ± 0.017
PLA-T	1.252	1.209 ± 0.012
A10-U	1.261	1.174 ± 0.011
A10-T	1.267	1.186 ± 0.027
A20-U	1.277	1.091 ± 0.006
A20-T	1.282	1.114 ± 0.037
A30-U	1.293	0.772 ± 0.015
A30-T	1.298	0.773 ± 0.015
C10-U	1.261	1.169 ± 0.035
C10-T	1.267	1.165 ± 0.019
C20-U	1.277	1.067 ± 0.062
C20-T	1.283	1.091 ± 0.070
C30-U	1.294	0.858 ± 0.041
C30-T	1.299	0.859 ± 0.041
P10-U	1.260	1.152 ± 0.024
P10-T	1.266	1.159 ± 0.019
P20-U	1.274	1.072 ± 0.032
P20-T	1.280	1.064 ± 0.014
P30-U	1.290	0.678 ± 0.004
P30-T	1.295	0.674 ± 0.019

**Table 6:** Water absorption (% mass uptake) of rotomolded PLA biocomposites at different immersion times, for untreated (U) and annealed (T) samples. Values are expressed as mean ± standard deviation (*n* = 5).

Sample	Immersion Time					
	2 h	24 h	7 d	14 d	21 d	28 d
PLA-U	0.42 ± 0.02	0.81 ± 0.05	1.12 ± 0.01	1.14 ± 0.09	1.11 ± 0.10	1.13 ± 0.07
PLA-T	0.30 ± 0.01	0.61 ± 0.02	0.66 ± 0.04	0.84 ± 0.06	0.85 ± 0.04	0.84 ± 0.09
A10-U	1.29 ± 0.03	3.35 ± 0.21	6.75 ± 0.44	7.01 ± 0.22	7.30 ± 0.21	7.35 ± 0.33
A10-T	1.20 ± 0.07	2.50 ± 0.07	5.61 ± 0.12	7.23 ± 0.21	7.29 ± 0.17	7.29 ± 0.20
A20-U	2.42 ± 0.21	6.68 ± 0.25	11.15 ± 0.17	11.29 ± 0.13	11.58 ± 0.10	11.86 ± 0.16
A20-T	2.03 ± 0.10	6.61 ± 0.12	10.80 ± 0.16	13.11 ± 0.06	13.08 ± 0.28	13.13 ± 0.26
A30-U	11.70 ± 0.98	19.60 ± 1.26	26.08 ± 2.41	25.44 ± 1.66	25.43 ± 0.68	25.81 ± 1.19
A30-T	9.80 ± 0.56	20.94 ± 0.94	36.58 ± 0.27	41.96 ± 1.48	41.69 ± 2.01	41.21 ± 0.79
C10-U	0.74 ± 0.02	2.15 ± 0.11	5.24 ± 0.22	5.61 ± 0.22	6.14 ± 0.08	6.37 ± 0.29
C10-T	1.15 ± 0.06	2.43 ± 0.08	4.67 ± 0.28	6.09 ± 0.05	6.46 ± 0.03	6.60 ± 0.06
C20-U	1.66 ± 0.09	5.38 ± 0.19	9.87 ± 0.27	10.59 ± 0.08	11.14 ± 0.13	11.68 ± 0.10
C20-T	2.00 ± 0.07	5.63 ± 0.27	10.09 ± 0.16	12.48 ± 0.10	13.27 ± 0.22	13.32 ± 0.31
C30-U	7.95 ± 0.39	16.44 ± 0.76	20.06 ± 0.92	20.31 ± 1.01	20.35 ± 0.57	20.13 ± 2.41
C30-T	5.16 ± 0.07	14.74 ± 0.39	21.25 ± 0.59	27.24 ± 0.96	27.48 ± 1.77	27.50 ± 1.63
P10-U	1.22 ± 0.02	2.60 ± 0.10	5.08 ± 0.05	5.29 ± 0.11	5.79 ± 0.11	6.16 ± 0.10
P10-T	1.10 ± 0.02	2.34 ± 0.05	5.48 ± 0.07	6.74 ± 0.07	7.00 ± 0.38	6.99 ± 0.28
P20-U	0.93 ± 0.04	2.59 ± 0.08	7.86 ± 0.23	8.79 ± 0.21	9.18 ± 0.17	9.63 ± 0.12
P20-T	1.57 ± 0.02	3.66 ± 0.13	10.55 ± 0.16	12.52 ± 0.36	12.96 ± 0.43	12.94 ± 0.73
P30-U	9.02 ± 0.53	15.91 ± 0.77	19.38 ± 0.38	19.71 ± 0.53	19.74 ± 0.98	20.88 ± 1.16
P30-T	8.36 ± 0.35	19.25 ± 1.26	28.59 ± 0.16	32.88 ± 0.85	37.14 ± 1.81	37.77 ± 2.44

**Table 7:** Carter–Kibler [51] two-stage model parameters for the water absorption of neat PLA untreated (PLA-U) and annealed (PLA-T) samples.

Sample	*M_∞_* (%)	*D* (mm^2^/s)	*α* (s^−1^)	*β* (s^−1^)	*R* ^2^
PLA-U	1.13	2.43 × 10^−6^	1.01 × 10^−4^	6.82 × 10^−5^	0.9997
PLA-T	1.03	1.46 × 10^−7^	5.02 × 10^−5^	5.46 × 10^−5^	0.9868

**Table 8:** Disintegration (% mass loss) of rotomolded PLA biocomposites at different lab-scale composting times, for untreated (U) and annealed (T) samples. Values are expressed as mean ± standard deviation (*n* = 4).

Sample	Mass Loss (%) at Selected Composting Times			
	7 d	14 d	21 d	28 d
PLA-U	2.69 ± 0.17	21.08 ± 2.69	26.26 ± 2.84	54.37 ± 2.88
PLA-T	4.12 ± 0.03	19.91 ± 1.55	31.96 ± 3.72	35.71 ± 1.82
A10-U	3.61 ± 0.18	26.97 ± 1.21	49.29 ± 1.28	53.82 ± 3.85
A10-T	7.48 ± 0.52	29.68 ± 4.29	47.10 ± 1.33	51.52 ± 1.72
A20-U	3.09 ± 0.01	21.75 ± 1.55	37.76 ± 1.69	47.38 ± 1.44
A20-T	6.28 ± 0.33	22.04 ± 1.12	31.14 ± 1.90	69.58 ± 5.50
A30-U	9.31 ± 0.86	27.78 ± 1.73	50.49 ± 2.04	65.07 ± 4.62
A30-T	25.62 ± 2.79	32.64 ± 0.68	33.97 ± 1.88	74.43 ± 5.70
C10-U	3.34 ± 0.09	24.04 ± 2.91	35.94 ± 0.13	56.82 ± 5.90
C10-T	5.30 ± 0.44	27.47 ± 1.16	29.14 ± 1.87	38.93 ± 2.27
C20-U	4.30 ± 0.25	22.49 ± 0.51	26.65 ± 2.67	33.53 ± 1.31
C20-T	4.43 ± 0.14	21.02 ± 1.53	21.95 ± 0.38	28.14 ± 2.83
C30-U	1.40 ± 0.12	16.21 ± 1.44	31.04 ± 2.39	45.05 ± 3.06
C30-T	16.83 ± 0.75	25.41 ± 2.09	38.94 ± 3.02	55.60 ± 2.15
P10-U	4.65 ± 0.08	13.21 ± 0.99	19.85 ± 0.65	22.17 ± 0.47
P10-T	6.48 ± 0.36	15.84 ± 0.90	22.26 ± 2.04	54.16 ± 3.81
P20-U	5.67 ± 0.26	25.20 ± 3.90	40.04 ± 2.38	60.23 ± 4.26
P20-T	9.49 ± 0.32	27.01 ± 3.81	30.91 ± 1.92	57.02 ± 6.05
P30-U	8.65 ± 0.81	43.70 ± 3.15	50.56 ± 0.40	65.55 ± 0.13
P30-T	69.60 ± 0.99	92.63 ± 2.87	100.00 ± 0.00	100.00 ± 0.00

## Data Availability

The data presented in this study are available from the corresponding author upon reasonable request.

## Abbreviations

The following abbreviations are used in this manuscript:

ASTM	American Society for Testing and Materials
CrI	Crystallinity index
DSC	Differential scanning calorimetry
FWHM	Full width at half maximum
ISO	International Organization for Standardization
PLA	Poly(lactic acid)
SEM	Scanning electron microscopy
XRD	X-ray diffraction
